# Optical Biosensing System for the Detection of Survivin mRNA in Colorectal Cancer Cells Using a Graphene Oxide Carrier-Bound Oligonucleotide Molecular Beacon

**DOI:** 10.3390/nano8070510

**Published:** 2018-07-09

**Authors:** Katarzyna Ratajczak, Bartlomiej E. Krazinski, Anna E. Kowalczyk, Beata Dworakowska, Slawomir Jakiela, Magdalena Stobiecka

**Affiliations:** 1 Department of Biophysics, Warsaw University of Life Sciences (SGGW), 159 Nowoursynowska Street, 02776 Warsaw, Poland; katarzyna_ratajczak@sggw.pl (K.R.); beata_dworakowska@sggw.pl (B.D.); 2 Department of Human Histology and Embryology, University of Warmia and Mazury, 30 Warszawska Street, 10082 Olsztyn, Poland; bartlomiej.krazinski@uwm.edu.pl (B.E.K.); a.kowalczyk@uwm.edu.pl (A.E.K.)

**Keywords:** graphene oxide nanosheet nanocarrier, hairpin–hairpin interactions, survivin mRNA, molecular beacon probe, SW480 cancer cells

## Abstract

The anti-apoptotic protein survivin is one of the most promising cancer biomarkers owing to its high expression in human cancers and rare occurrence in normal adult tissues. In this work, we have investigated the role of supramolecular interactions between a graphene oxide (GO) nanosheet nanocarrier and a survivin molecular beacon (SurMB), functionalized by attaching fluorophore Joe and quencher Dabcyl (SurMB-Joe). Molecular dynamics simulations revealed hydrogen bonding of Joe moiety and Dabcyl to GO carriers that considerably increase the SurMB-GO bonding strength. This was confirmed in experimental work by the reduced fluorescence background in the OFF state, thereby increasing the useful analytical signal range for mRNA detection. A new mechanism of hairpin–hairpin interaction of GO@SurMB with target oligonucleotides has been proposed. A low limit of detection, LOD = 16 nM (S/N = 3), has been achieved for complementary tDNA using GO@SurMB-Joe nanocarriers. We have demonstrated an efficient internalization of SurMB-Joe-loaded GO nanocarriers in malignant SW480 cells. The proposed tunability of the bonding strength in the attached motifs for MBs immobilized on nanocarriers, via structural modifications, should be useful in gene delivery systems to enhance the efficacy of gene retention, cell transfection and genomic material survivability in the cellular environment.

## 1. Introduction

The interactions of diagnostic and therapeutic oligonucleotides with nanocarriers used for their delivery have recently been the focus of theranostic research [1]. The strength of these interactions and transport modality provide the framework for gene retention [1,2,3] and protection while in transport, and facilitate their controlled release. From among the various nanocarriers studied for delivery systems, including gold nanoparticles [4,5,6], liposomes [7,8,9,10], micelles [11], exosomes [12], graphene oxide [2,13,14,15,16,17,18,19] and others [20,21], herein we focus specifically on the binding of molecular beacon (MB) probes to graphene oxide (GO) nanosheet nanocarriers and the mechanism of hybridization of the GO-bound molecular beacon (GO-MB) with target strands. Recently, we have demonstrated the ability of a hairpin MB to interact with a hairpin-structured target oligonucleotide [8], thus extending the Tyagi–Kramer model [22] of MB interaction with linear oligonucleotides. Therefore, the mechanism considered here is the hairpin–hairpin interaction of GO-bound MB with target strands, followed by the duplex DNA desorption from GO nanocarriers [23,24,25,26]. The aim is to investigate the feasibility of tuning the GO-MB binding strength to protect MBs against nucleases, while enabling unhindered antisense oligonucleotide release and hybridization with mRNA in the cytosol of cells.

The use of MBs allows not only the detection and imaging of specific mRNAs overexpressed in cancer cells, but also enables the prevention of their post-transcriptional translation to unwanted proteins, such as the proliferation enhancing and anti-apoptotic AIP-group proteins [27,28]. The oligonucleotide drugs are usually administered in the form suitable to act as a short interfering RNA (siRNA) or micro-RNA (miRNA), which are non-coding RNAs able to exert gene silencing action on mRNA via complex biochemical pathways [29]. While the use of siRNA and miRNA is being studied extensively, it is evident that an alternative way based on capturing mRNA using MB oligonucleotides in target-controlled delivery is also a viable solution for cancer treatment. 

Survivin (Sur) is an anti-apoptotic and proliferation enhancing protein [30] and member of an apoptosis inhibitor protein (AIP) family, which is overexpressed in cancer cells [31]. The expression of survivin mRNA and the survivin protein in colorectal cancer was recently investigated using molecular biology methods, including reverse transcription-polymerase chain reaction (RT-PCR) [32,33], Western blot [33] and ELISA [34] assays, as well as immunohistochemical staining [35].

In this work, we have employed a molecular beacon-based optical biosensing system as an alternative method for the detection of survivin mRNA in colorectal cancer cells. Colorectal cancer is one of the three most common cancers in the world. The survivin molecular beacon (SurMB) probe used in this study consisted of a single-stranded oligonucleotide with a Joe fluorescence dye and Dabcyl quencher attached to the 5′ and 3′ ends of the strand, respectively. The loop of the probe was an antisense oligonucleotide targeting survivin mRNA. The interactions of this SurMB-Joe probe with a GO nanocarrier (GO@SurMB-Joe) have been studied to determine if there are any supramolecular forces able to bind the Joe or Dabcyl moieties to a GO carrier, which have never been investigated before. These interactions are important for holding the probe on a nanocarrier and protecting it against digestion by nucleases [36]. The molecular dynamics simulations (MD) were performed to gain new insight into the supramolecular interactions of SurMB-Joe with GO nanocarriers, and its basic operation principles. In this study, the mechanism of the hairpin–hairpin interaction of the GO-bound SurMB-Joe with target oligonucleotides was investigated for the first time, following the single-nucleotide polymorphism sensitivity determination. In vitro studies of intracytoplasmic Sur mRNA detection in SW480 colorectal cancer cells using GO@SurMB-Joe nanoprobes were also performed.

## 2. Results and Discussion

### 2.1. Hairpin Structures of SurMB-Joe, tDNA Target, and the Hybridization Product

**Figure 1:**
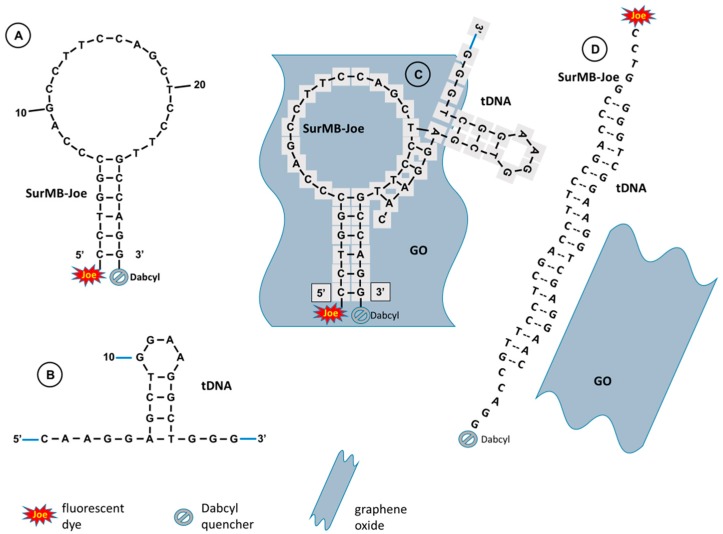
Structures and sequences of (**A**) survivin molecular beacon with a fluorophore Joe (SurMB-Joe) and (**B**) target oligonucleotide (tDNA) complementary to loop of MB; (**C**,**D**) Principle of molecular beacon operation, interactions of SurMB-Joe with GO and tDNA.

The ability of a hairpin MB to interact with a hairpin-structured target oligonucleotide (tDNA) has recently been demonstrated [8], and a hairpin–hairpin interaction model was proposed to extend the Tyagi–Kramer model [22] of molecular beacon interactions with a linear target oligonucleotide. Our calculations—performed using the UNAFold software—indicate that the target oligonucleotide (tDNA), complementary to the loop of SurMB, preferentially forms a stable small-loop hairpin structure instead of relaxing to a linear conformation. Figure 1A presents the sequence and the most thermodynamically stable hairpin structure of SurMB-Joe, with negative Gibbs free energy of formation ΔG°_SurMB-Joe_ = −4.26 kcal/mol [37]. The loop sequence of the probe was composed of an antisense oligonucleotide targeting the survivin mRNA region [38]. Figure 1B presents the sequence and hairpin structure of tDNA with formation energy ΔG°_tDNA_ = −2.33 kcal/mol. The general schemes of the principle of SurMB-Joe operation and its interactions with tDNA and graphene oxide (GO) nanosheet nanocarriers are depicted in Figure 1C,D. According to our model, in the first step, the hybridization of one leg of tDNA with molecular beacon loop occurs (Figure 1C), followed by the melting of a target oligonucleotide stem and, finally, full hybridization of tDNA with the SurMB-Joe loop. Upon the hybridization process, the opening of the molecular beacon structure takes place, followed by desorption of the formed DNA duplex from the surface of GO carriers, restoring the fluorescence signal of Joe dye (Figure 1D). Since GO is such a strong quencher, the duplex DNA must be desorbed before its fluorescence is restored. The adsorption/desorption processes of linear DNA onto/from a GO platform were studied recently by Wu et al. [23] and Park et al. [3], but the conformation and melting temperatures have not yet been considered. On the other hand, the attachment of a small quenching graphite nanoparticle (NP) to the end of a MB strand was applied by Piao et al. [36]. Although the use of GO is gaining popularity in DNA sensing applications [39], the exact conformation of DNA strands has rarely been evaluated [37].

### 2.2. Supramolecular Interactions of SurMB-Joe Components with GO Nanocarriers

**Figure 2:**
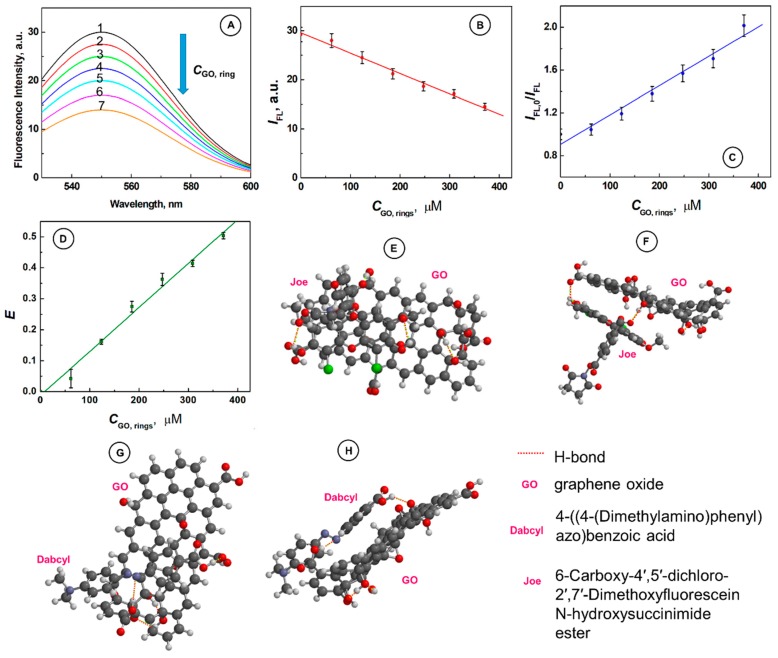
(**A**) Fluorescence spectra for SurMB-Joe after addition of GO at different concentrations *C*_GO,rings_ (µM): (1) 0, (2) 61.8, (3) 123.5, (4) 185.4, (5) 247.3, (6) 308.9, (7) 380.8; (**B**) dependence of *I*_FL_ vs. *C*_GO,rings_; (**C**) Stern–Volmer plot of *I*_FL,0_/*I*_FL_ vs. *C*_GO,rings_; (**D**) dependence of quenching efficiency *E* on *C*_GO,rings_; (**E**) top and (**F**) side views of GO-Joe supramolecular structures with H-bonds marked with red dashed lines; (**G**) top and (**H**) side views of GO-Dabcyl supramolecular structures with H-bonds marked with red dashed lines. Joe: 6-Carboxy-4′,5′-dichloro-2′,7′-dimethoxyfluorescein N-hydroxysuccinimide ester; Dabcyl: 4-((4-(Dimethylamino)phenyl) azo) benzoic acid. Conditions: *C*_SurMB_ = 100 nM; buffer: 10 mM PBS, pH 7.4; room temp.

At the room temperature and in the absence of a complementary target oligonucleotide, SurMB-Joe remains in the OFF conformation with very low residual fluorescence intensity due to the close proximity of the Joe fluorophore to the Dabcyl quencher. Upon the interaction with a GO carrier, which acts as a supplementary quencher, a further reduction of the already low fluorescence of SurMB-Joe is observed, as seen in Figure 2. The intensity decreases from *I*_FL,1_ = 29.2 a.u. to *I*_FL,1_ = 14.5 a.u. after addition of GO to the level of 370 µM (GO rings), as illustrated in Figure 2A,B. On the basis of these data, the Stern–Volmer quenching constant was determined using the dependence:

*I*_FL,0_/*I*_FL_ = 1 + *K*_SV_*Q*, (1)

where *I*_FL,0_ and *I*_FL_ are the fluorescence intensities of SurMB-Joe (the donor) at the emission maximum *λ*_max_ = 548 nm, in the absence and presence of GO nanocarriers (acting as the acceptors), respectively; *K*_SV_ is the Stern–Volmer quenching constant, and *Q* is the GO quencher concentration. The value of *K*_SV_ obtained from the slope of the plot of *I*_FL,0_/*I*_FL_ vs. *Q* = *C*_GO,rings_, presented in Figure 2C, is: *K*_SV_ = 2742 ± 21 M^−1^. This relatively high value of *K*_SV_ indicates on the static quenching mode. Figure 2D presents the plot of quenching efficiency *E* vs. *C*_GO,rings_. The quenching efficiency was calculated from the dependence:

*E* = 1 − *I*_FL_/*I*_FL,0_ (2)

To confirm the modality of the quenching interactions, we considered if the collisional frequency of molecules involved is high enough to achieve the observed high quenching efficiency. In the specific case of GO nanosheets, the mobility of GO is practically null, so the only factor influencing the collisional frequency is associated with the MB colliding with GO. However, with the concentration of MB in the nanomolar range, the frequency of collisions is so low [40] that the probability of dynamic quenching becomes insignificant. Moreover, single-stranded oligonucleotides are known to assemble on GO, which points to static—rather than dynamic—quenching. Furthermore, we considered supramolecular forces between the fluorophore Joe and GO, which also indicates the static quenching mode.

To evaluate what kind of interactions between the fluorophore Joe and a GO nanosheet carrier may cause static quenching of Joe, we have performed molecular dynamics and quantum mechanical calculations for ensembles of GO@Joe. As shown in Figure 2E,F, up to two hydrogen bonds (marked with red dashed lines) can be formed between the Joe molecule and a GO nanosheet. In Figure 2F, a stacking configuration of the supramolecular ensemble GO@Joe is also shown. Hence, the ability of Joe to form supramolecular ensembles with GO nanosheets is consistent with the static quenching mode.

Supramolecular interactions of Joe with GO nanocarriers are important to the overall MB binding to GO, as they increase the total MB binding strength. We have also investigated if the Dabcyl quencher can contribute to the binding strength of MBs to GO nanocarriers. The molecular dynamics and quantum mechanical calculations performed for ensembles of GO@Dabcyl have revealed that supramolecular interactions do occur. In Figure 2G–H, the formation of hydrogen bonding between Dabcyl and GO is shown. It is seen that one hydrogen bond is established between the nitrogen of Dabcyl and the hydrogen of the carboxylic group of GO, and another hydrogen bond is formed between the hydrogen of the carboxylic group of Dabcyl and the oxygen of the carboxylic group of GO. The stacking configurations of GO@Dabcyl supramolecular structures are also viable, as shown in Figure 2H. This means that both the fluorophore Joe and quencher Dabcyl contribute to the total binding strength of the MB oligonucleotide to the GO nanocarrier, in addition to the MB's single-stranded DNA contribution. The enhancement of the overall binding strength of MB to the GO nanocarrier by supramolecular interactions of Joe fluorophore and Dabcyl quencher to the GO nanocarrier is a significant development which improves the efficiency of the genomic material delivery to cells for diagnostic, imaging, and therapeutic purposes. The increased strength of MB binding to GO also results in the reduced fluorescence in the OFF state, thereby increasing the useful analytical signal for mRNA detection.

### 2.3. Desorptive Hybridization of GO-Bound SurMB-Joe with Complementary tDNA Target and Mutants

**Figure 3:**
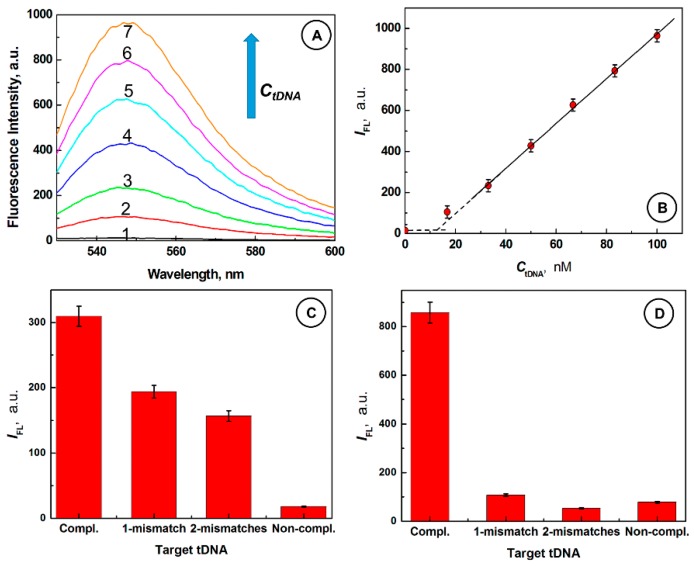
(**A**) Fluorescence spectra for SurMB-Joe@GO ensembles after addition of complementary *t*DNA with different concentrations, *C_t_*_DNA_ (nM): (1) 0, (2) 16.7, (3) 33.3, (4) 50, (5) 66.7, (6) 83.3, (7) 100; (**B**) Dependence of fluorescence peak intensity *I*_FL_ vs. *C*_tDNA_; (**C**,**D**) Comparison of fluorescence peak intensities for SurMB-Joe@GO ensembles upon addition of a complementary target, targets with 1- and 2-mismatches, and non-complementary oligonucleotides at the temperatures: (**C**) 37 °C and (**D**) 61 °C; Conditions: *C*_SurMB_ = 100 nM; *C*_GO,ring_ = 370.8 µM; buffer: 10 mM PBS, pH 7.4, room temp. (**A**,**B**), temperature scan from 22 °C to the indicated temperature (**C**,**D**).

Figure 3A shows that the fluorescence signal of GO@SurMB-Joe ensembles increase from *I*_FL,1_ = 14.5 a.u. in the absence of tDNA, to *I*_FL,7_ = 963.1 a.u. after 30 min of interaction with a 100 nM complementary tDNA (curves 1 and 7, respectively). Figure 3B shows the dynamic range for complementary tDNA determination, with the detection limit of 12 nM, from the intersection of lines, and 16 nM, using the standard three-sigma method for complementary target oligonucleotides. The strong fluorescence observed upon addition of a complementary target tDNA strand is due to the formation of a stiff, open conformation of the SurMB-tDNA duplex, warranting large separation between the fluorophore Joe and quencher Dabcyl, and subsequent detachment of the SurMB-tDNA duplex from the GO surface. The control experiments using mismatched and noncomplementary oligonucleotides were also performed at different temperatures. The results are depicted in Figure 3C,D. It is seen in both cases that the interactions of GO@SurMB-Joe ensembles with mismatched and non-complementary targets are much weaker than those with a complementary target. At 37 °C, the fluorescence signals for targets with one and two mismatches are 193.7 and 156.9 a.u., respectively, which are considerably lower than the fluorescence signal observed upon addition of a complementary oligonucleotide (309.5 a.u.). At 61 °C, fluorescence observed upon addition of mismatched target oligonucleotides decreased below 100 a.u., due to melting of weak associations between GO@SurMB and targets, while that for a complementary target increased to over 800 a.u., providing an excellent discrimination against the mutants. These results indicate that the GO@SurMB-Joe probe exhibits a single-nucleotide polymorphism sensitivity.

### 2.4. Detection of Survivin mRNA in Colorectal Cancer Cells SW480 Using GO@SurMB-Joe Nanoprobes

In further studies, we have investigated the transfection of SW480 cells with GO@SurMB-Joe to assess the feasibility of applications of these nanocarriers for gene delivery and detection of survivin mRNA in colorectal cancer cells.

**Figure 4:**
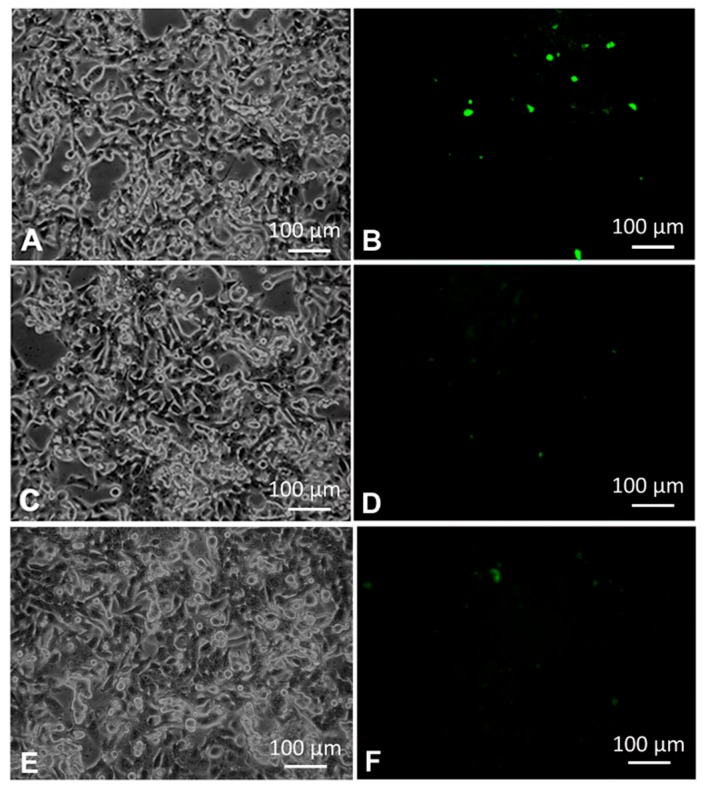
Optical (left panel) and fluorescence (right panel) images of SW480 cells: (**A**,**B**) cells transfected with GO@SurMB-Joe; (**C**,**D**) cells transfected with GO alone; (**E**,**F**) cells transfected with SurMB-Joe; transfection time: 4 h.

The optical and fluorescence microscopy images obtained for the SW480 cell line are presented in Figure 4A. Successful transfection of SW480 cells with GO@SurMB-Joe and detection of Sur mRNA is clearly seen in Figure 4B. The green fluorescence image shows that after 4 h of incubation with GO@SurMB-Joe, the fluorescence signal appears in the cytoplasm of SW480 cells. It indicates that SurMB-Joe has been internalized in the cancer cells and, due to the strong expression of survivin mRNA in these cells, the fluorescence emission from the Joe fluorophore could be observed. The imaging was made possible by the hybridization of the molecular beacon with specific regions of the survivin mRNA sequence, followed by a disassembly of the formed SurMB-Joe-mRNA duplex from the GO carrier. The efficiency of transfection was analyzed using luminosity histograms. The histograms presented the number of pixels (counts) vs. luminosity within the green channel. The total number of pixels analyzed (total counts) was ca. 5.665 × 10^6^ per image. The spectrum for SW480 cancer cells transfected via the GO platform shows lower luminosity than that of a standard for SW480 cells with Lipofectamine, which affords 50% transfection efficiency. Therefore, assuming this efficiency as the standard, we can estimate the internalization efficiency for the GO@SurMB, by comparing the obtained experimental luminosity values.

In the negative tests performed, the transfection of SW480 cells with bare graphene oxide nanocarriers (Figure 4D) and with molecular beacon alone (Figure 4F) resulted in negligible fluorescence signal in comparison to that obtained for GO@SurMB-Joe carriers (Figure 4B). These experiments show that graphene oxide nanocarriers at 67 ng/mL concentration make it a feasible platform for cancer diagnostics and gene delivery. At concentrations of GO higher than ca. 17 µg/mL, the inner filter effects interfere with measurements of fluorescence emission, and therefore other analytical methods would need to be employed to assess the feasibility of using higher nanocarrier doses. The inner filter effect observed at higher concentrations of GO is due to the high absorption of UV–Vis light by the GO nanocarriers, leading to the decrease of the intensity of the excitation beam in measurements of SurMB fluorescence in comparison to the case of the absence of GO. Results of the experiments described above validate the GO nanocarriers ability to transfer an oligonucleotide payload through a cell membrane, as well as corroborate the hairpin–hairpin interaction model [8] for GO-bound MBs in desorptive hybridization with hairpin mRNA targets, which is an extension of the original Tyagi–Kramer model developed for MBs interacting with linear oligonucleotide targets.

### 2.5. Mechanism of Hairpin–Hairpin Interactions in Desorptive Hybridization of GO@SurMB-Joe and tDNA

**Figure 5:**
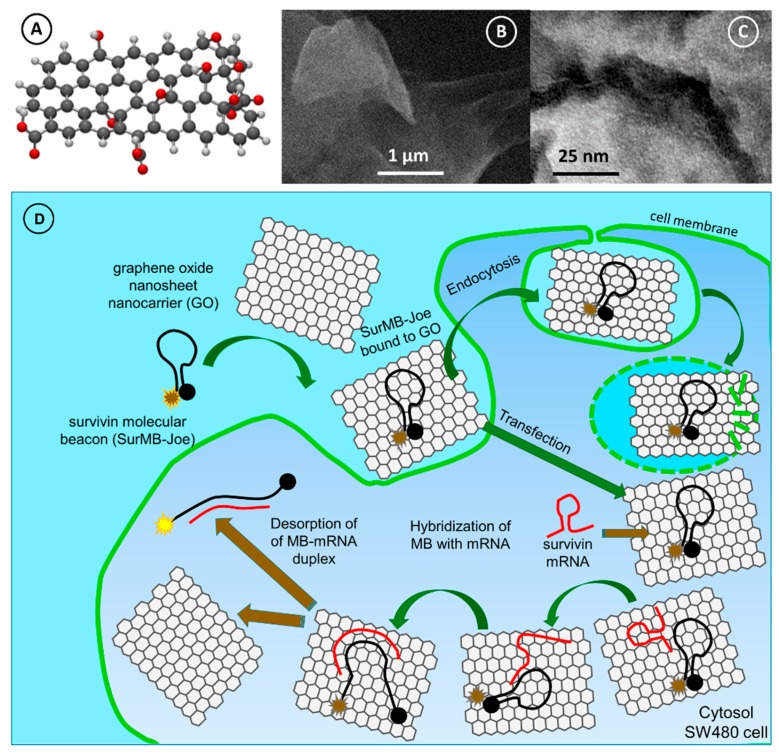
(**A**) Structure of a model graphene oxide nanosheet nanocarriers (GO); (**B**) SEM image of GO nanocarriers; (**C**) TEM image of GO; (**D**) Principle of the internalization of the GO-bound SurMB-Joe (GO@SurMB-Joe) to SW480 cells.

In Figure 5, the structure and morphology of graphene oxide nanocarriers used in experiments is presented. The model structure of a GO nanosheet nanocarrier, with the following functional groups: three carboxylic groups (–COOH), four hydroxyl groups (–OH) and three carbonyl groups (–C=O), is presented in Figure 5A. From the scanning electron microscopy (SEM) images (Figure 5B), it is seen that the GO nanosheets have a flake-like shape with rippled structure. The transmission electron microscopy (TEM) images show a wrinkled and corrugated morphology of GO.

Figure 5D presents an overview of the cellular uptake of GO carriers loaded with SurMB-Joe (GO@SurMB-Joe) into malignant SW480 cells. In this image, the hybridization of SurMB-Joe with survivin mRNA, present in cytosol of cancer cells, is depicted, following the hairpin–hairpin interaction principle. The opening of the molecular beacon structure is initiated upon this hybridization, and it is followed by the formed duplex DNA/RNA desorption from the surface of a GO carrier, restoring the fluorescence signal of a Joe dye. Endocytosis is the most frequently occurring mode of internalization, and occurs when membrane receptors recognize compounds which are required by the cell. Alternatively, internalization by transfection is also well-known and widely utilized, such as in gene delivery applications. This mode of internalization proceeds when an oligonucleotide is bound to a transfer molecule which is able to pass through the membrane and, thus, can carry the genomic material into the cytosol through the membrane. Due to their high affinity to lipids, graphene oxide nanocarriers are able to interact directly with the membrane, thereby permitting the passing of their cargo through the membrane. This happens at low concentrations of GO nanocarriers, as used in this work, at ca. 65 ng/mL. The high affinity of GO to lipid membranes has recently been confirmed for high concentrations of GO [41]. When GO concentrations are high (>10 µg/mL, up to 200 µg/mL), GO will embed itself in the membrane and may even cause the formation of a pore in the membrane. On the basis of these considerations, and to cover the functionalization of GO with poly-L-lysine (PLL), under study, which we have already investigated for modifying gold nanoparticles for gene delivery via transfection, we consider the transfer of SurMBs by the GO nanocarriers as proceeding mainly via the transfection mechanism, but with the possibility of endocytosis. In the case of endocytosis, the liposomes which carry the GO@SurMBs will be rapidly perforated by GO drawing up the lipids from the liposome membrane, with the remaining part of the pathway occurring in the same way as in the case of transfection. Please note that liposomes tightly encasing GO@SurMB cannot survive long enough to trap the nanocarrier since GO interacts with the lipid membrane and draws the lipids, destroying the tiny membrane. The above "gelum" model is not exploring the problem of complex intracellular distribution of survivin mRNA, signaled by Bao and colleagues [42], but rather attempts to emphasize the process of hairpin–hairpin interaction of the target mRNA with the GO-bound MB, which has not been considered before. The importance of complex intracellular distribution of Sur mRNA warrants further studies, including colocalization experiments with lysotracker and other markers, to verify the complex intracellular trafficking of genomic material. This trafficking is particularly important in cancer metastasis driven by intercellular communication [12], whereby the genomic material is exported from the cancer cells via exosomes to infect or reprogram distant healthy cells. As a result, the complex intracellular trafficking of genomic material is the subject of ongoing studies in our labs. Furthermore, the model discussed above does not take into account the fact that the real cellular membrane tension is heterogeneous [43]. The heterogeneity of membrane tension is due to the lipid flow resistance associated with the net of cytoskeleton-bound transmembrane proteins, which make the drug-carrying nanocarrier internalization uneven over the membrane surface.

The schematic in Figure 5D includes both the transfection and endocytosis as parallel pathways, and which pathway will be predominant in a given system will depend on the details of the nanocarrier functionalization. For instance, functionalization of GO with poly-l-lysine (PLL) will make transfection predominant, while pegylation may favor the endocytosis mechanism. After adding a complementary target oligonucleotide to the well-quenched GO@SurMB-Joe assemblies, a large increase in fluorescence intensity is observed. This is consistent with the interaction mechanism that we have encountered in recent studies of molecular beacon delivery nanocarriers for detection of survivin mRNA in U-87 MG human malignant glioma cells [2], whereby a single-stranded oligonucleotide immobilized on GO nanocarriers hybridized with the complementary target, forming a duplex which then desorbed from the nanocarrier (Figure 1D and Figure 5D).

## 3. Materials and Methods

### 3.1. Chemicals

The survivin molecular beacon (SurMB), which is an antisense oligonucleotide-targeting survivin mRNA with a sequence of 5′-Joe-*CCTGGC* CCA GCC TTC CAG CTC CTT *GCCAGG*-Dabcyl-3′ (SurMB-Joe), and the oligonucleotide complementary to the loop of SurMB-Joe (S_t_), with a sequence of 5′-CAA GGA GCT GGA AGG CTG GG-3′, were synthesized by the Laboratory of DNA Sequencing and Oligonucleotides Synthesis, Institute of Biochemistry and Biophysics of the Polish Academy of Sciences (IBB PAS, Warsaw, Poland) and FutureSynthesis (Poznan, Poland), respectively. The purity of these oligonucleotides was tested by high-performance liquid chromatography (HPLC). Single-layer graphene oxide nanosheet nanocarriers (GO) dispersed in water were purchased from ACS Materials, LLC (Medford, MA, USA). All chemicals were of analytical grade purity. Aqueous solutions were prepared with freshly deionized water with 18.2 MΩ cm resistivity (Hydrolab Sp. z o.o. Sp.K., Straszyn, Poland). All concentrations of added reagents cited in this paper are final concentrations obtained after mixing.

### 3.2. Apparatus

The fluorescence spectra were recorded using Spectrometer model LS55 (Perkin Elmer, Waltham, MA, USA), with 20 kW pulsed Xenon light source and a photomultiplier tube detector. The excitation and emission slit widths were set to 5.0 nm and scan speed to 500 nm/min. The measurements were performed in 10 mM PBS buffer, pH 7.4. The excitation wavelength was set to *λ*_ex_ = 520 nm. The fluorescence cell images were acquired with a Nikon Eclipse TE300 inverted light microscope with a blue B-2A fluorescence filter with a 30–50 nm bandwidth excitation filter, long-pass dichromatic mirror and long-pass barrier filter. Images were recorded digitally using a Canon Power Shot A640 scope-mounted camera. All images were made with exactly the same exposure. They were then exported to Photoshop Elements and the brightness and contrast of all images were adjusted using the Levels function for green channel by shifting the black limit from 0 to 26 of the green luminosity scale which effectively enabled removing the trace background luminosity. The molecular dynamics (MD) simulations of interactions of GO with Joe and Dabcyl were performed using Spartan 14 software (Wavefunction, Irvine, CA, USA). The calculations of MB and target nucleotide structures, their folding energies, and melting temperatures were performed using the University of Albany web server DINAMelt providing the program UNAFold ver. 3.9 with a Quikfold application (RNA Institute, University of Albany, Albany, NY, USA).

### 3.3. Cell Culture

The human colon cancer cell line SW480 was purchased from ATCC (LGC Standards Sp. z.o.o., Lomianki, Poland) and was cultured in culture medium containing Dulbecco’s modified Eagle’s Medium (DMEM) supplemented with 10% fetal bovine serum (FBS), in a humidified atmosphere of 5% CO_2_ in the air at 37 °C using a Shell Lab Model 2123-TC CO_2_ Incubator (Cornelius, OR, USA). The SW480 cells were subcultured every 2–3 days. After experiments, the used cells were collected and disposed appropriately.

### 3.4. Cell Transfection

Transfection experiments with SW480 cells were conducted with graphene oxide nanocarriers (GO@SurMB-Joe). In this case, 268 μL of 100 ng/mL (3.7 µM, rings) GO solution was mixed with 1 μL of 100 μM SurMB-Joe and 131 μL of DMEM and added to the cells, followed by 4 h incubation.

## Supplementary Materials

Details of chemicals and the graphene oxide nanosheet nanocarriers concentration, in terms of the concentration of graphene rings, are available online at http://www.mdpi.com/2079-4991/8/7/510/s1.
