# Supplementary material for: Optical Biosensing System for the Detection of Survivin mRNA in Colorectal Cancer Cells Using a Graphene Oxide Carrier-Bound Oligonucleotide Molecular Beacon
Source: Nanomaterials (Basel). 2018 Jul 9;8(7):510. doi: 10.3390/nano8070510 (PMC6071027; doi:10.3390/nano8070510)
Supplement: Supplementary file 1 [file nanomaterials-08-00510-s001.pdf]

Optical Biosensing System for the Detection of Survivin mRNA in Colorectal Cancer Cells using Graphene Oxide Carrier–Bound Oligonucleotide Molecular Beacon

Katarzyna Ratajczak[1], Bartlomiej E. Krazinski[2], Anna E. Kowalczyk[2], Beata Dworakowska[1], Slawomir Jakiela[1*] and Magdalena Stobiecka[1*]

[1] Department of Biophysics, Warsaw University of Life Sciences (SGGW), 159 Nowoursynowska Street, 02776 Warsaw, Poland

[2] Department of Human Histology and Embryology, University of Warmia and Mazury, 30 Warszawska Street, 10082 Olsztyn, Poland

* Correspondence: Magdalena Stobiecka, e-mail: magdalena_stobiecka@sggw.pl; Tel: +48-22-593-8614; Slawomir Jakiela, e-mail: slawomir_jakiela@sggw.pl; Tel: +48-22-593-8626

MATERIAL AND METHODS

Chemicals

Survivin molecular beacon (SurMB-Joe) was antisense oligonucleotide targeting survivin mRNA with sequence 5’-Joe - CCTGGC CCA GCC TTC CAG CTC CTT GCCAGG - Dabcyl-3’ and oligonucleotide complementary to loop of SurMB-Joe (tDNA), with sequence 5’-CAA GGA GCT GGA AGG CTG GG-3’ have been synthesized by the Laboratory of DNA Sequencing and Oligonucleotides Synthesis, Institute of Biochemistry and Biophysics of the Polish Academy of Sciences (IBB PAS, Warsaw, Poland) and FutureSynthesis (Poznan, Poland), respectively. The purity of these oligonucleotides was tested by HPLC. Single layer graphene oxide nanosheet nanocarriers (GO) dispersed in water with thickness 0.6-1.2 nm and single-layer ratio >80%, were purchased from ACS Materials, LLC (Medford, MA, USA). Sodium phosphate dibasic (Na_2HPO_4) and potassium phosphate monobasic (KH_2PO_4) were obtained from Sigma-Aldrich Chemical Company (St. Louis, MO, USA). The human colon cancer cell line SW480 was purchased from ATCC (LGC Standards Sp. z.o.o., Lomianki, Poland). Fetal bovine serum (FBS), L-glutamine, penicillin/streptomycin and Dulbecco’s modified Eagle’s Medium (DMEM) were obtained from cell culture company PAA (Immuniq, Warsaw, Poland). Aqueous solutions were prepared with freshly deionized water with 18.2 MΩ cm resistivity (Hydrolab Sp. z o.o. Sp.K., Straszyn, Poland). All concentrations of added reagents cited in this paper are final concentrations obtained after mixing.

Concentration of graphene rings

The molar mass of a graphene ring was assumed from the following equation published in our earlier work [1]:

M(graphene ring) = $n_C \alpha_C M_C$ (1)

where n_C is the number of carbon atoms in the graphene ring, α_C is the fraction of each carbon atom belonging to the given ring, and M_C =12 g/mol is the atomic mass of carbon.

Thus, M = 6*(1/3)*12 = 24 g/mol.

The content of oxygen in graphene oxide has been determined to be p = 11 weight %, so the molar mass of an average GO ring is approximately: $M_{GO,rings}$ = 24/(1 - p/100) = 26.97 g/mol. Thus, C_{GOs} = 1 mg/mL = (1/26.97) M = 37.08 mM (of GO rings).

1. Ratajczak, K.; Stobiecka, M., Ternary Interactions and Energy Transfer Between Fluorescein Isothiocyanate, Adenosine Triphosphate, and Graphene Oxide Nanocarriers. *J. Phys. Chem. B* 2017, 121, 6822−6830.
